# Revisiting the Effects of High-Speed Railway Transfers in the Early COVID-19 Cross-Province Transmission in Mainland China

**DOI:** 10.3390/ijerph18126394

**Published:** 2021-06-13

**Authors:** Chun-Hsiang Chan, Tzai-Hung Wen

**Affiliations:** Department of Geography, National Taiwan University, Taipei 10617, Taiwan; d04228002@ntu.edu.tw

**Keywords:** transfer service, COVID-19, Wuhan city lockdown, high-speed rail network, intercity population flow, spatial transmission

## Abstract

Coronavirus disease 2019 (COVID-19) is an ongoing pandemic that was reported at the end of 2019 in Wuhan, China, and was rapidly disseminated to all provinces in around one month. The study aims to assess the changes in intercity railway passenger transport on the early spatial transmission of COVID-19 in mainland China. Examining the role of railway transport properties in disease transmission could help quantify the spatial spillover effects of large-scale travel restriction interventions. This study used daily high-speed railway schedule data to compare the differences in city-level network properties (destination arrival and transfer service) before and after the Wuhan city lockdown in the early stages of the spatial transmission of COVID-19 in mainland China. Bayesian multivariate regression was used to examine the association between structural changes in the railway origin-destination network and the incidence of COVID-19 cases. Our results show that the provinces with rising transfer activities after the Wuhan city lockdown had more confirmed COVID-19 cases, but changes in destination arrival did not have significant effects. The regions with increasing transfer activities were located in provinces neighboring Hubei in the widthwise and longitudinal directions. These results indicate that transfer activities enhance interpersonal transmission probability and could be a crucial risk factor for increasing epidemic severity after the Wuhan city lockdown. The destinations of railway passengers might not be affected by the Wuhan city lockdown, but their itinerary routes could be changed due to the replacement of an important transfer hub (Wuhan city) in the Chinese railway transportation network. As a result, transfer services in the high-speed rail network could explain why the provinces surrounded by Hubei had a higher number of confirmed COVID-19 cases than other provinces.

## 1. Introduction

For a respiratory infectious disease, person-to-person contact plays a vital role in the transmission of an epidemic, and a large population flow raises the probability of contact between people [1,2,3,4,5,6]. An imported case of the disease could trigger a serious local outbreak. Border control policies could be an effective measure to prevent imported cases from causing local outbreaks. Therefore, recent studies have focused on assessing the effectiveness of national border control policies in containing the domestic and global spread of respiratory infectious diseases, such as body temperature screening in airports and comprehensive travel history investigations [7,8,9,10,11,12,13]. Some studies have found evidence that supports this approach because it can delay the peak of the pandemic and reduce the number of confirmed cases; however, some have argued that the efficiency of disease control is still limited [14]. The primary reason is that the implementation of border control is too slow and that local transmission has already occurred by the time it is implemented. Another reason is that the accuracy of positive detection during border screenings is too low to efficiently differentiate infected persons because fever is not a consistent symptom of influenza, or symptoms may develop after arrival [15,16,17]. For instance, only 13.8% of infected people were detected in Australia during the SARS epidemic [18], and only 6.6% of imported cases were detected in Japan during the H1N1 period [17].

Due to its high transmissibility, COVID-19 is an ongoing pandemic that was reported at the end of 2019 in Wuhan, China [19,20,21] and rapidly disseminated to all provinces in around one month [22]. The virus was primarily spread through the massive annual cross-province migration during the Spring Festival from 24 to 30 January in 2020 [23]. The Spring Festival travel season, also referred to as Chunyun, is from 10 January to February 18 in 2020 [24]. During this period, the demand for domestic transport (railways, air transportation, and bus systems) reaches its peak [25]; furthermore, railways are one of the most crucial transport modes because of their high accessibility and low cost. The city of Wuhan, which is a critical transfer hub in Chinese railway transport, has two primary intercity rails (the Jingguang railway and the Shanghai–Wuhan–Chengdu passenger railway) connected to most of the major cities (e.g., Beijing, Shanghai, and Guangzhou) [26,27]. In consideration of both the severe situation of COVID-19 and the hub characteristics during Chunyun, the Chinese government enacted emergency measures in Wuhan city and the surrounding cities and counties of Hubei Province on 23 January effective from 25 January to 8 April in order to restrict all population outflows, control pandemic transmission, and expand social distancing. Hence, the Wuhan city lockdown might have seriously affected population flows in railway transport.

Recent studies have assessed the impacts of the Wuhan city lockdown on pandemic transmission through time and space. By simulating different levels of travel reduction from Hubei or Wuhan, researchers could estimate the spatial progress of the pandemic within neighboring provinces and overseas airports before and after the Wuhan city lockdown [28]. These studies have pointed out that greater reductions in travel efficiently decreased the development of the pandemic in the surrounding provinces or countries connected by roads or flights. Moreover, some studies have also indicated that city lockdown policies could delay the timing of the infection peak [29,30,31,32,33,34]. In other words, city lockdowns controlled pandemic transmission and bought time for the healthcare system to address the outbreaks [24,35]. Due to Wuhan’s status as an important railway hub in mainland China, the Wuhan city lockdown has reduced many infections because of the resultant sizable cross-province population flow changes. These studies have indicated that the provinces bordering Hubei had a higher number of confirmed cases than other provinces, even after the Wuhan city lockdown [24]. However, the possible reasons of a large number of confirmed cases concentrated in the neighboring provinces of Hubei have not been fully discussed. Therefore, this study aims to assess the changes in railway passenger transport on the early spatial transmission of COVID-19 in mainland China. Daily railway schedule data were used in this study to measure intercity population mobility patterns in order to capture the impact of the Wuhan city lockdown. Examining the role of railway transport properties in disease transmission could help quantify the spatial spillover effects of large-scale travel restriction interventions, such as city lockdowns. It could improve our understanding of the spatial diffusion patterns of COVID-19 during early transmission in mainland China.

## 2. Data

### 2.1. Railway Schedule Data

To compare the changes in railway transportation before and after the Wuhan city lockdown, we collected the daily train timetable from the official Chinese railway reservation website (https://www.12306.cn/index/, accessed on 22 January 2020) by web crawler from 22 to 26 January 2020. Each train’s unique key is collected daily from the official Chinese train reservation website; then, the complete train timetable of the specific train could be fetched by the unique key and save to the local train timetable database. There are 12 columns within train timetable data, including the day of arrival (e.g., today or tomorrow), station name, train type, base station indicator (1 represents the first station, while “NaN” indicates other stations), station name of the final destination, the arrival time of the station, base station name, train code, the day offset of arrival (0 means the train will arrive today, while 1 indicates the train will arrive tomorrow), departure time of this station, station index (i.e., the station index of this train), and duration (from the previous station to this station). There are approximately 8540 timetables for each day. The daily schedule data cover 3129 stations in 325 cities. Four fields for each record are used in this study, including the names of the departure and destination stations, the name of the station stops along the route, and the train code. The Chinese railway includes 10 different train types (G, C, D, Z, T, K, S, L, Y, and N). Train types G, C, and D are cross-province transport with high-speed trains; Z, T, and K are intercity transport with lower-speed trains; and the rest of the train types are within-city transport. In this study, we focused on the cross-province movement of people; therefore, two major types of high-speed trains were incorporated into the following analyses: the D-series and G-series high-speed trains. (Train types G and C are of the same train type; therefore, we combined them into one train type, G.) Within the dataset collected, 32% and 42% of trains are G-Series and D-Series High-Speed Train each day, respectively; furthermore, G-Series and D-Series High-Speed Train cover 960 stations and 256 cities.

We further transformed the daily train tables into city-to-city origin-destination (OD) networks to visualize and analyze the spatial patterns of intercity railway transport. The nodes of the OD network represent each city, and the link weights represent the frequency with which trains travel between those cities. We obtained the differences in train frequency before and after the Wuhan city lockdown (22 January/26 January 2020) to represent the changes in intercity passenger movement due to the large-scale travel restriction policy.

### 2.2. COVID-19 Cases

**Figure 1:**
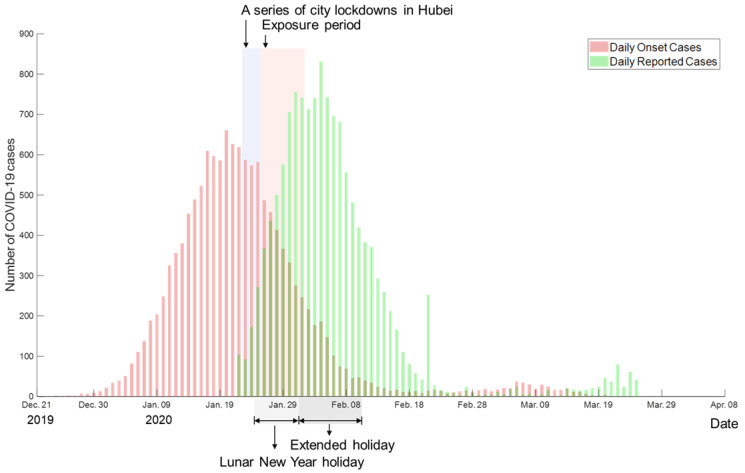
The timeline of pandemic transmission in mainland China (Hubei province was excluded).

The reported date for each confirmed COVID-19 case was collected from summary reports from the National Health Commission of China [36]. The data collection period ranges from 22 January 2020 to 25 March 2020. To depict the temporal epidemic progression in the early transmission period, we adopted the study conducted by Li, Guan, Wu, Wang, Zhou, Tong, Ren, Leung, Lau, Wong, Xing, Xiang, Wu, Li, Chen, Li, Liu, Zhao, Liu, Tu, Chen, Jin, Yang, Wang, Zhou, Wang, Liu, Luo, Liu, Shao, Li, Tao, Yang, Deng, Liu, Ma, Zhang, Shi, Lam, Wu, Gao, Cowling, Yang, Leung, and Feng [19]) to estimate the date of symptom onset for each confirmed case from symptom onset distributions and reported date distributions in Figure 1.

## 3. Methods

In this cross-sectional study, we used a daily train timetable (960 stations) to observe the changes in intercity passenger movement (256 cities) before and after the Wuhan city lockdown. Two network properties, PageRank and betweenness centrality were used to quantify the structural changes in the intercity railway OD network after the Wuhan city lockdown. The spatial distribution of intercity transportation network properties was characterized by bivariate k function and k-nearest neighbor statistic. Eight province-level socioeconomic indicators were considered as confounders. Bayesian multivariate regression was used to examine the association between structural changes in the railway OD network and the incidence of COVID-19 cases.

### 3.1. Structural Changes in Intercity Railway Transport

Wuhan is an important railway hub during Chunyun; thus, the Wuhan city lockdown substantially affected the cross-province population flow. This could be reflected in the structural changes to the intercity railway OD network. We used network centrality metrics to measure the nodal properties of the OD network, including PageRank centrality, which represents the destination arrival probability (DAP), and betweenness centrality, which reflects the potential for transfer activity (PTA) for each city.

PageRank centrality (*PR*) measures the probability that a person randomly clicks on a particular link from anywhere; hence, a higher value indicates higher importance [37,38]. The definition of the PR of a particular city $C_{i}$ is as follows:

(1) $$PR\left(C_{i}\right)=\underset{C_{j}\in M\left(C_{i}\right)}{\sum }\frac{PR\left(C_{j}\right)}{L\left(C_{j}\right)},$$

where $M\left(C_{i}\right)$ is the set of cities that connect to city $C_{i}$, and $L\left(C_{j}\right)$ is the number of outbound connections with city $C_{j}$. The recursive equation starts with rank $PR\left(C_{j}\right)$ and then stops the iteration when it converges. PageRank represents the staying probability of a particular city when starting from any city in the railway OD network. Thus, we use this metric as an indicator to evaluate the DAP of the city. A higher value of the DAP represents a higher probability of staying in that specific city [39].

Betweenness centrality (*BC*) indicates the ratio of the number of shortest paths through a particular city to the total number of shortest paths between two given cities in the entire city network as follows [40,41]:

(2) $$BC\left(C_{i}\right)=\underset{k\neq i\neq j\in N}{\sum }\sigma_{kj}\left(i\right)/\sigma_{kj},$$

where $\sigma_{kj}$ is the total number of shortest paths between city $C_{k}$ and city $C_{j}$, and $\sigma_{kj}\left(i\right)$ is the number of shortest paths through a particular city $C_{i}$. Betweenness centrality is adopted to measure the PTA of the city because a higher betweenness centrality value stands for a higher number of transfers passing through the city [42,43].

We then used spatial statistics to compare the spatial patterns in the nodal metrics (PageRank and betweenness centrality) before and after the Wuhan city lockdown. A city with rising nodal metrics represents that city becoming a more influential node in the railway OD network after the Wuhan city lockdown. In contrast, a city with declining nodal metrics represents that city becoming a less influential node. The bivariate k function, a spatially summarized statistic, is used to evaluate the spatial clustering of cities with declining nodal metrics around cities with rising ones [44]. The k-nearest neighbor (kNN) statistic is used to assess the spatial proximity between cities with rising and declining nodal metrics [45,46].

### 3.2. The Association between Intercity Transportation Network Properties and the Number of Confirmed COVID-19 Cases

The changes in nodal metrics in the railway OD network could alter the contact probabilities within intercity population flows, which may influence the temporal progression of the COVID-19 epidemic. The changes in nodal metrics, PageRank, and betweenness centrality were aggregated into the province level, as were the destination arrival and transfer service statistics, respectively. Bayesian multivariate regression was used to measure the province-level association between the changes in the nodal metrics and the incidence of COVID-19 cases. The province-level socioeconomic indicators are incorporated as control variables in the regression model. These confounders include three dimensions: demographics, economics, and healthcare conditions. The variables in the demographics dimension include the total household population, the resident population percentage (the percentage of people who live in a specific area for six months or more), and the percentage of the household population living in other provinces (the proportion of the difference between the total population and the resident population). The variables in the economics dimension include the average gross domestic product (GDP) per capita. The variables in the healthcare conditions dimension consist of life expectancy, average local health expenditure per person, birth insurance coverage, and average birth insurance expenditure. All data are from the National Bureau of Statistics of China [47,48,49].

To avoid collinearity among the predictor variables, principal component analysis (PCA) was conducted to reduce the dimensions of the socioeconomic indicators by extracting the principal components. PCA is a statistical method used to obtain principal components from observations through orthogonal transformation. The first principal component (PC) can be regarded as the greatest amount of variance that can explain the most variance in the observations. To prevent the biased estimation of model parameter coefficients due to a small sample size (*n* = 28), a Bayesian multivariate regression is applied to estimate the effect of changes in nodal metrics on the incidence of confirmed COVID-19 cases by controlling for the PCs of the socioeconomic indicators. PCA and the Bayesian linear regression were performed by the R packages BAS 1.5.5 and STATS 3.6.2.

## 4. Results

### 4.1. Descriptive Statistics

**Figure 2:**
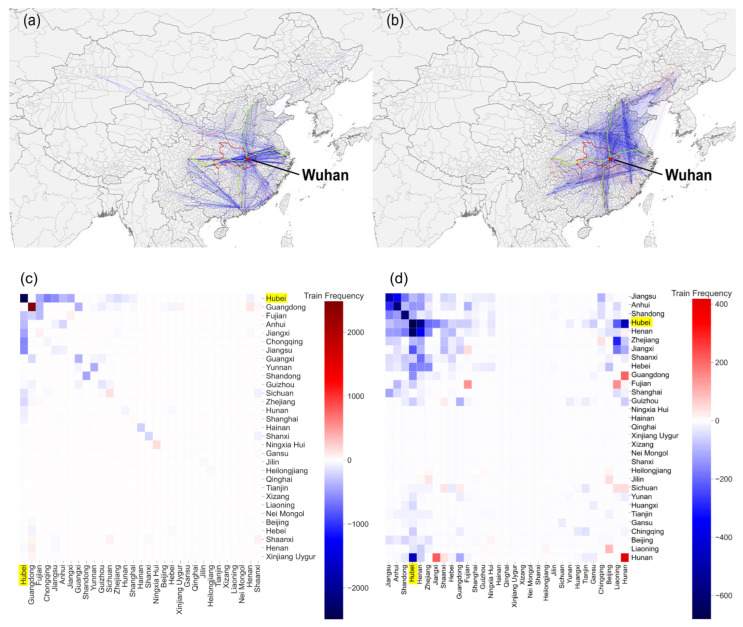
The changes in train frequency in the OD network after the Wuhan city lockdown at the city and province levels, where (**a**) D-series and (**b**) G-series high-speed trains are shown at the city scale. Blue represents decreased train frequency, and red represents increased frequency. The longitudinal railway is the Jingguang railway (green line), and the widthwise railway is the Shanghai–Wuhan–Chengdu railway (green line). The province-level changes for the (**c**) D-series and (**d**) G-series high-speed trains are shown to identify the routes most affected by the Wuhan city lockdown.

Figure 2 shows the daily number of G-series and D-series high-speed trains that stopped in Wuhan/Hubei, ranging from 19 January to 2 February 2020. When the city lockdown took effect, the number of each type of train dropped sharply. Within the dataset used, each day, 32% and 42% of trains are G-series and D-series high-speed trains, respectively; furthermore, G-series and D-series high-speed trains cover 960 stations in 256 cities. A significant decrease in the frequency of D-series trains centered on Wuhan city and extended widthwise (e.g., toward Shanghai, Jiangsu, Anhui, Hubei, Chongqing, and Sichuan) and in longitudinal directions (e.g., toward Beijing, Henan, Hubei, Hunan, and Guangdong), which is shown in Figure 2a. The frequency of trains between Guangdong and its surrounding provinces (e.g., Fujian, Yunnan, Guizhou, and Sichuan) also decreased. For G-series trains (Figure 2b), the decreasing pattern extended in a longitudinal direction (Beijing, Shandong, and Fujian) and two widthwise directions (Shanghai, Henan, and Shaanxi; and Zhejiang, Hunan, and Guizhou). Figure 2c shows that the Wuhan city lockdown mainly affected Hubei, Jiangsu, Chongqing, Jiangxi, and Anhui for the D-series high-speed trains. The major provinces affected by the G-series high-speed trains were Hubei, Henan, Shandong, Anhui, and Jiangsu (Figure 2d).

### 4.2. The Changes in Nodal Metrics

**Figure 3:**
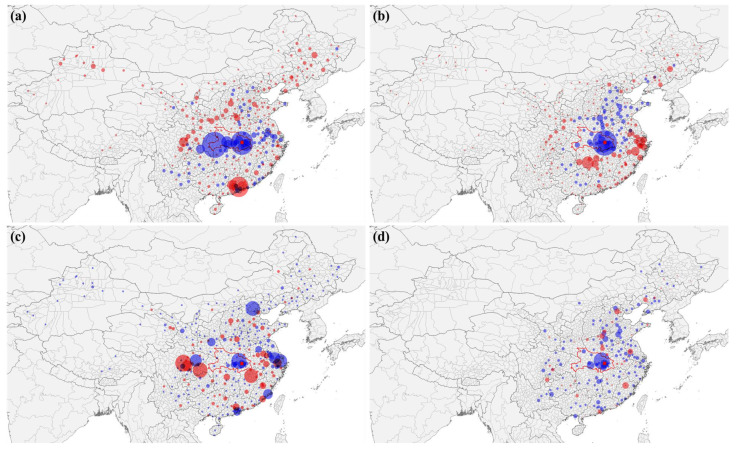
The changes in DAP and PTA after the Wuhan city lockdown. The declining and rising nodal metrics are illustrated by blue and red dots, respectively. The dot size represents the magnitude of the changes in nodal metrics. (**a**) The DAP changes in D-series trains; (**b**) the DAP changes in G-series trains; (**c**) PTA for D-series trains; (**d**) PTA changes for G-series trains.

Figure 3a,b shows the spatial patterns in the changes in DAP. The declining DAP pattern occurs in the major cities lying on the widthwise Shanghai–Wuhan–Chengdu railway (see Figure 2a,b) for the D-series trains. For the G-series trains, a declining DAP pattern occurs in the longitudinal cities along the Jingguang railway (see Figure 2a,b). The declining DAP pattern in the G-series and D-series is consistent with the train frequencies in the railway OD network and shows that the cities with rising DAP surround the cities with declining DAP. Figure 3c,d shows that neither the declining nor rising PTA patterns for the G-series and D-series trains follow a railway route. Most of the cities with declining PTAs are primary cities in mainland China, such as Beijing, Shanghai, and Guangdong. Additionally, the spatial distribution of cities with declining and rising PTA has a pattern similar to that of the distribution of declining and rising DAP.

### 4.3. Spatial Patterns in the Nodal Metrics Before and After the Wuhan City Lockdown

**Figure 4:**
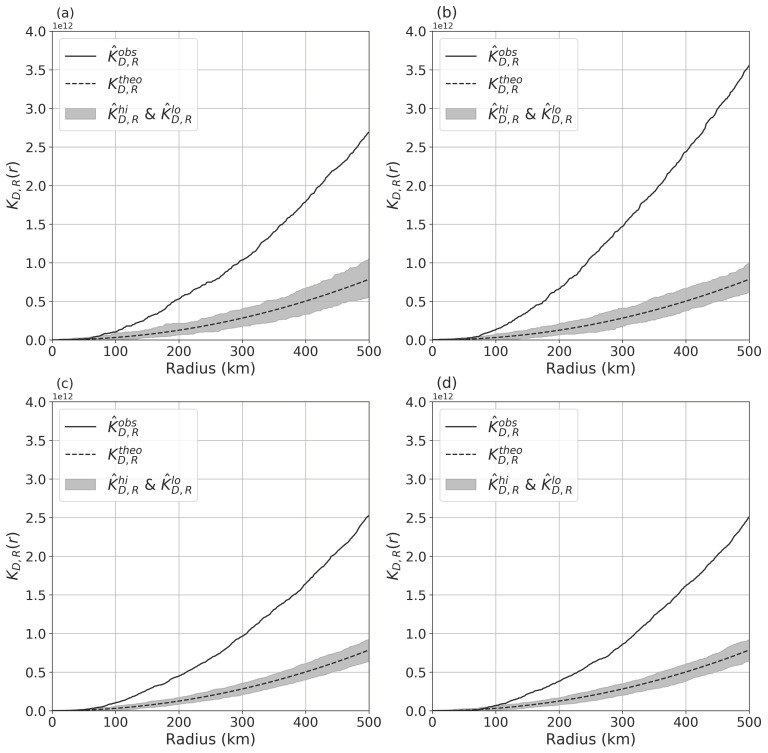
The results of the bivariate k function representing a significant spatial clustering tendency of cities with declining railway network properties around cities with rising properties. The network properties include the potential for transfer activity (PTA) for (**a**) D-series high-speed trains and (**b**) G-series high-speed trains and destination arrival probability (DAP) for (**c**) D-series high-speed trains and (**d**) G-series high-speed trains.

**Figure 5:**
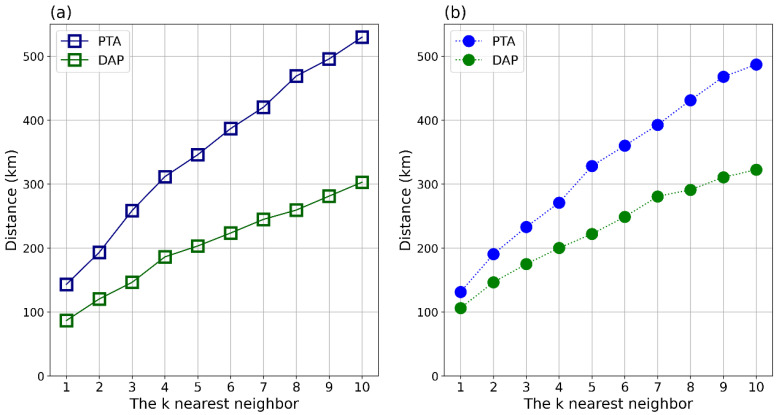
Spatial proximity of cities with declining railway network properties to cities with rising properties, where k varies from one to ten. (**a**) The square lines are the D-series high-speed trains, and (**b**) the circle lines are the G-series high-speed trains. Blue is PTA, and green is DAP.

Figure 4 represents the results of bivariate spatial analysis, showing spatial clustering patterns of the nodal metrics. It indicates that the cities with declining nodal metrics were significantly clustered near the cities with rising metrics for all types of high-speed trains and nodal metrics. Then, the median distance between the k-nearest city with rising railway network properties and that with declining railway network properties is presented in Figure 5. The results show that the distance between the two types of cities was larger when considering changes in PTA than changes in DAP for both types of high-speed trains, and they show that the extent of the impact on transfer services is geographically larger than that on destination arrivals. Taking D-series high-speed trains as an example, the distances from the cities with declining DAP and PTA to the 3rd nearest city with rising DAP and PTA were 146.2 km and 258.5 km, respectively. The gray area is the 95% confidence interval according to the $K_{D,R}^{theo}$ value.

### 4.4. The Effect of Changes in Nodal Metrics on COVID-19 Transmission

#### 4.4.1. Spatial Distribution of Nodal Metrics

**Figure 6:**
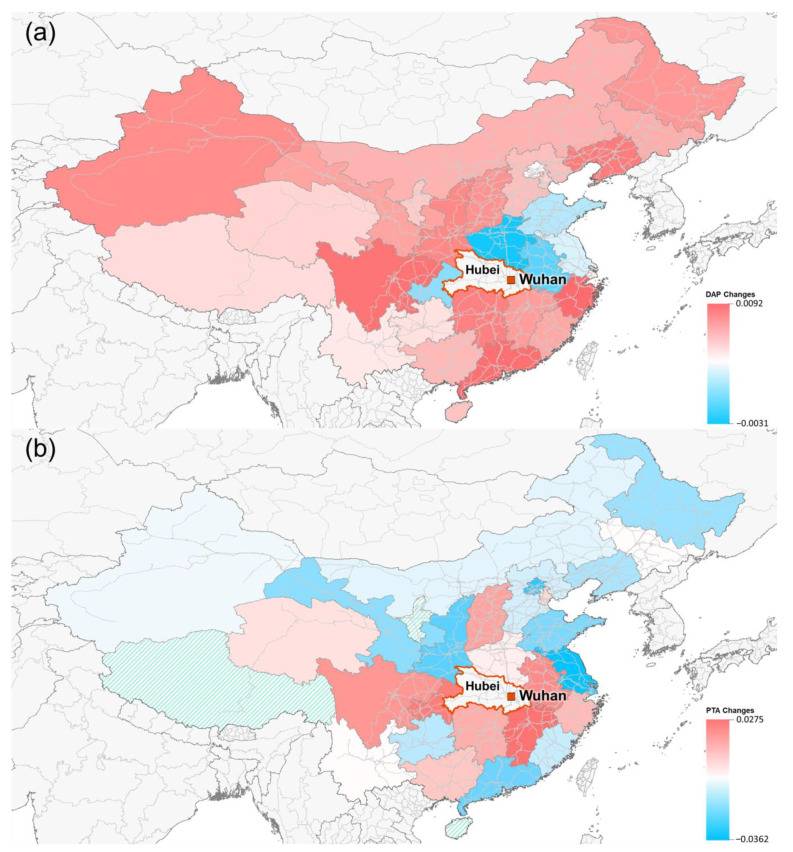
The spatial distribution of the provinces with changes in nodal metrics: (**a**) DAP and (**b**) PTA. The red provinces indicate those with rising nodal metrics; the blue provinces indicate those with declining nodal metrics.

Figure 6a,b shows the spatial changes in destination arrival (DAP) and transfer service (PTA) after the Wuhan city lockdown. These results show that after the Wuhan city lockdown, most provinces had rising DAP, and the provinces with rising PTA were around Hubei Province, including the widthwise provinces from Zhejiang to Qinghai and the longitudinal provinces from Shanxi to Guangxi. This result indicates that the Wuhan city lockdown caused rising destination arrival and high levels of transfer service in neighboring provinces during the early COVID-19 transmission stages.

#### 4.4.2. Clarification of the Role of the Frequency of Transfer Activities in COVID-19 Transmission

**Figure 7:**
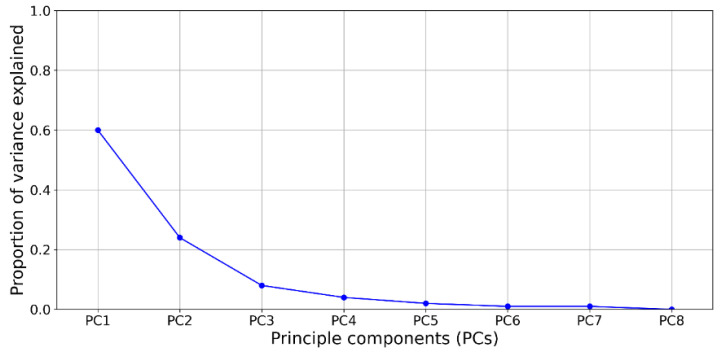
The proportion of variance explained by each principal component.

Figure 7 shows the proportion of variance explained by eight PCs obtained by PCA. The first three PCs were selected for our regression model because they explain 92% of the variance. According to the variable loadings in Table 1, the first principal component (PC1) was mainly dominated by the resident population percentage. The second principal component (PC2) was driven by the average local health expenditure per person. The total household population and life expectancy represented the third principal component (PC3). Hence, PC1, PC2, and PC3 captured the dimensions of floating populations, healthcare availability, and general demographics, respectively.

The Bayesian regression models evaluate the impact of destination arrival and transfer services on the incidence of confirmed COVID-19 cases, as shown in Table 2 and Table 3, respectively. The R^2^ of destination arrival and transfer services models are 0.49 and 0.60, respectively; thus, the transfer services model could explain more proportion of variance than the destination arrival one. The results indicate that destination arrival service does not have a significant impact on the incidence of confirmed COVID-19 cases after controlling for the PCs of the socioeconomic indicators; however, transfer service does have a significantly positive effect on early COVID-19 transmission after the Wuhan city lockdown. In other words, transfer activities could promote the spread of COVID-19. Our results also reveal that healthcare availability has a negative effect on the incidence of confirmed cases, while the total population of the province (PC3) has a positive effect (negative regression coefficient and negative PC variable loadings), which means a large population could be regarded as a risk factor for disease transmission. Corresponding to the results of spatial analyses, the neighboring provinces of Hubei with a high number of confirmed cases have a high transfer service score, including Anhui, Jiangxi, Hunan, and Chongqing. The provinces with a low average local health expenditure per person, a short life expectancy, a high household population result in a high number of confirmed cases, such as Henan, Guangdong, Hunan, Zhejiang, Shandong, Jiangsu, and Sichuan. Nevertheless, the top five provinces (Henan, Anhui, Guangdong, Jiangxi, and Hunan) with severe COVID-19 epidemics are captured via this model.

## 5. Discussions

High-speed trains can be regarded as a major tool used for cross-province transportation during Chunyun in mainland China. Therefore, high-speed railway schedules could capture cross-province movement patterns. With limited data sources, including COVID-19 reported cases from open data platform and daily high-speed railway schedule by the web crawler, this study compared the differences in city-level network properties (destination arrival and transfer service) before and after the Wuhan city lockdown in the early stages of the spatial transmission of COVID-19 in mainland China. Our results show that the regions with increasing transfer activities had significant numbers of confirmed infected cases, and these regions were located in provinces neighboring Hubei in the widthwise and longitudinal directions. These results indicate that transfer activities enhance the probability of interpersonal transmission and could be a crucial risk factor for increasing epidemic severity after the Wuhan city lockdown. Our study provides another possible pathway to explain why the provinces surrounded by Hubei had a higher number of confirmed COVID-19 cases than other provinces.

In pandemic transmission, considering the network connectivity of a person is crucial because it could reflect the frequency or probability of that person contacts others. Hence, several network indicators have been widely used to measure the network connectivity characteristics of each person and have revealed the associations between network connectivity measures, such as degree centrality, PageRank, and betweenness centrality, and pandemic transmission [50,51,52,53,54]. Degree centrality is used to measure the number of persons connected to the specific person, and so a high value indicates that this person most likely infected others because he or she can reach more people than others can. Previous studies have reported that a high level of degree centrality is positively related to disease incidence [51,55]. However, degree centrality only includes the first-degree neighbors connected to the specific person, and it cannot account for other neighbors who are not directly connected to that person. PageRank takes account not only of all the people in the network but also of the direction and weight of the connection between one person and another [53]. Betweenness centrality represents the mediation property of each person in the network. Previous studies have reported that people with high mediation have greater infection potential due to increased contact with various people [56,57]. Different from previous studies, our results show no relationship between the changes in PageRank centrality and early COVID-19 transmission, but the changes in betweenness centrality are significantly related to the incidence of confirmed COVID-19 cases after the Wuhan city lockdown. Two factors could explain this. First, the destinations for most railway passengers were tier 1 or 2 cities (as measured by the Chinese government’s official city ranking), such as Beijing, Guangdong, or Henan [28]. In addition, travelers usually targeted their hometowns as destinations during Chunyun. Hence, transfer service changes could better explain early COVID-19 transmission than destination arrival service changes. Second, we measured changes in nodal metrics; if a region was not affected by the Wuhan city lockdown, it did not show significant changes. In other words, Wuhan and other cities in Hubei provided railway passengers with a transfer hub, not a destination; therefore, the Wuhan city lockdown did not affect the destination of railway passengers but changed their itinerary routes to their destination. As a result, understanding the effect of transfer services plays an essential role in understanding early COVID-19 transmission.

The definitions of PageRank and betweenness centrality, two network indicators, were used in this study to measure the properties of destination arrival [53,58] and transfer activities [39,42,43]. Our results show that the impact of transfer activities on early COVID-19 transmission was more significant than that of destination arrival. Some regions usually have few transfers; however, those regions provided more transfer services after the Wuhan city lockdown due to the closure of the critical transfer hub of Wuhan during Chunyun. The more people gathered in these regions, the higher the contact probability with various people becomes. A similar result for airport networks has been reported in Gardner and Sarkar [59]). They addressed the fact that transfer passengers are a vital element to monitor in order to avoid disease transmission, especially for airport surveillance. Uninfected passengers could be infected because they contacted infected people during transfers at the airport [6]. Our results further illustrate that the provinces neighboring Hubei had a rising PTA after the Wuhan city lockdown, and remarkably, individuals in these provinces had a greater probability of contact with infected persons than those in provinces with a declining PTA. This indicates that provinces neighboring Hubei provided partial transfer functionality after the Wuhan city lockdown and explains why those provinces had a larger number of confirmed cases.

The demographic, economic, and healthcare dimensions have been reported to play critical roles in pandemic transmission [48,49]. Our results show that transfer activities and the total population had positive impacts on COVID-19 transmission, whereas the resident population percentage and healthcare availability had negative impacts. A large population with a low resident population suggests that the spread of the infection might be driven by people who work in other regions. This implies that most of the workers and students returned to their hometowns during Chunyun, and the number of people and their contact probability in these regions quickly increased. Those provinces with a low GDP per capita, a low level of average birth insurance expenditure, and life expectancy have a higher number of infections, indicating that a lack of healthcare resources increased the incidence of confirmed cases [60]. Moreover, a high total household population might indicate high contact probabilities, while low average local government expenditures might indicate that more time is required to respond to the pandemic [61]. In addition to transfer activity, we further reveal that other risk factors in early COVID-19 transmission, such as a high total household population, low resident population, low GDP, low birth insurance expenditure, low average local government expenditure on healthcare, and short life expectancy, might increase pandemic transmission.

This study has several limitations. First, city-level socioeconomic indicators were not incorporated in our regression models. Thus, the characteristics of local transmission within a city could be overlooked in our study. The findings of this study reflect the impact of high-speed railway transport on cross-province COVID-19 transmission. Second, due to a lack of symptom onset data for each confirmed case, we estimated the symptom onset of cases from the published literature. This could lead to a biased temporal trend in estimates regarding early transmission in mainland China. Third, trains usually carry many passengers during Chunyun; therefore, the number of trains scheduled between two cities was used to represent population flows. It may not reflect the actual volume of passengers. Fourth, not only is high-speed rail transport important but air and road transport are also important cross-province transportation tools during Chunyun. Further investigation is warranted to incorporate more transport modes. Fifth, city-level transfer services were conducted through city-to-city network structures. These network indicators may not capture the actual behaviors of individual railway passengers. Sixth, although this study demonstrates the province-level association between transfer services and the COVID-19 epidemic in the early stages of transmission, it cannot infer individual-level infection risks from transfer behaviors. Last but not least, the spatial heterogeneity of population flow plays an important role in the geographical process of epidemic transmission. Therefore, it could be warranted to use geographically weighted regression to explore spatial heterogeneity of the COVID-19 epidemic in future studies.

## 6. Conclusions

The impact of the Wuhan city lockdown on railway transportation was measured by destination arrival and transfer activities using city-to-city network metrics, including the PageRank and betweenness centrality scores. Our results show that the provinces with rising transfer activities after the Wuhan city lockdown had more confirmed COVID-19 cases, but changes in destination arrival did not have significant effects. This implies that the destinations of railway passengers might not be affected by the Wuhan city lockdown, but their itinerary routes could be changed due to the replacement of an important transfer hub (Wuhan city) in the Chinese railway transportation network. We conclude that transfer services in the high-speed rail network could be another possible explanation for why the provinces surrounded by Hubei had a higher number of confirmed COVID-19 cases than other provinces.

## Figures and Tables

**Table 1:** The variable loadings for each principal component.

Confounding	PC1	PC2	PC3
Household population	0.09	−0.53	−0.77
Resident population percentage	0.42	−0.16	0.27
Percentage of the household population in other provinces	−0.42	0.16	−0.27
GDP per capita	−0.41	−0.24	0.11
Life expectancy	−0.26	−0.51	0.44
Average local health expenditure per person	−0.24	0.57	−0.13
Birth insurance coverage	−0.41	−0.16	−0.08
Average birth insurance expenditure	−0.42	−0.05	0.13

**Table 2:** The regression coefficients of destination arrival service and principal components of the socioeconomic indicators in the Bayesian multivariate model.

Variables	Mean	SD	2.5%	97.5%	*p* (*β* ≠ 0)
Intercept	96.07	12.13	71.69	121.48	1.00
Destination arrival service	−835.76	2532.82	−9101.57	1831.27	0.26
Resident population (PC1)	0.76	3.00	−3.84	9.78	0.24
Healthcare availability (PC2)	−34.72	8.85	−53.23	−16.92	1.00
General demographics (PC3)	−34.28	18.77	−64.21	0.00	0.88

Mean and SD indicate the average and standard deviation of the posterior distribution of each variable. P (*β* ≠ 0) is the marginal probability that a coefficient is nonzero.

**Table 3:** The regression coefficients of transfer service and principal components of the socioeconomic indicators in the Bayesian multivariate model.

Variables	Mean	SD	2.5%	97.5%	*p* (*β* ≠ 0)
Intercept	96.07	11.10	74.29	119.52	1.00
Transfer service	2025.33	1319.49	0.00	4160.11	0.82
Resident population (PC1)	−1.15	3.93	−13.27	5.00	0.31
Healthcare availability (PC2)	−36.71	8.54	−53.82	−20.02	1.00
General demongraphics (PC3)	−40.25	16.80	−65.75	0.00	0.94

Mean and SD indicate the average and standard deviation of the posterior distribution of each variable. P (*β* ≠ 0) is the marginal probability that a coefficient is nonzero.

## Data Availability

The data presented in this study are available on request from the corresponding author.

## Abbreviations

COVID-19: Coronavirus disease 2019; OD: Origin-destination; DAP: destination arrival probability; PTA: potential for transfer activity; PR: PageRank centrality; BC: Betweenness centrality; kNN: k-nearest neighbor; GDP: gross domestic product; PCA: principal component analysis.
